# Distinct underlying mechanisms contribute to learning-dependent distractor rejection

**DOI:** 10.3758/s13423-026-02997-2

**Published:** 2026-08-25

**Authors:** Brian A. Anderson

**Affiliations:** Department of Psychology, Texas A&M University, 4235 TAMU, College Station, TX 77843-4235 USA

**Keywords:** Selective attention, Selection history, Search efficiency, Spatial attention, Feature-based attention, Search guidance

## Abstract

The signal suppression hypothesis has been proposed as an explanation for why physically salient stimuli sometimes involuntarily draw attention and other times can be successfully ignored. Recent accounts of signal suppression, and learning-dependent distractor rejection more broadly, offer conflicting findings concerning the extent to which facilitated ignoring of a salient distractor is feature specific or feature general. The present study provides evidence for concurrent learning-dependent gains in feature-specific distractor rejection, which is sensitive to stimulus frequency and relatively short-lived, and feature-general distractor rejection, which builds from experience rejecting salient stimuli and is more enduring. On the first day of testing, participants were facilitated in ignoring salient distractors presented in a high-probability location and rendered in a high-probability color, which produced additive benefits, and were simultaneously better able to ignore salient distractors in general (including low-probability distractors) with practice. Critically, while the effects of the biased probabilities were no longer evident on the second day of testing in which these probabilities were equated, the effect of practice on ignoring remained and generalized to novel distractors. Together, these findings suggest that distinct underlying mechanisms capable of concurrently influencing information processing collectively give rise to the ability to more efficiently ignore salient distractors with experience.

## Introduction

Physically salient stimuli, such as the warning signals emitted by heavy construction vehicles as they move throughout a jobsite, are intuitively attention grabbing. However, salient signals have been shown to become far less attention grabbing with repeated exposure, reflecting a process of learning-dependent ignoring that can have devastating consequences in hazardous work settings (e.g., Anderson et al., 2024). The signal suppression hypothesis (e.g., Gaspelin & Luck, 2018b; Gaspelin et al., 2015, 2025; Luck et al., 2021) was formulated as a reproachment between the historically contentious ideas that physically salient stimuli are obligatorily attended (e.g., Theeuwes, 1992, 2010) and that whether such stimuli are attended is entirely contingent on goal-directed attentional control settings (e.g., Folk et al., 1992, 2002), and provides a straightforward explanation for the progressive blunting of salience-based attentional priority following repeated exposure to a salient signal. Specifically, the signal suppression hypothesis holds that physical salient stimuli generate a strong attentional priority signal that has the power to compel attention allocation, but this priority signal can be suppressed prior to attention being allocated to the salient stimulus, reducing or even eliminating the bias to selectively attend to it (Gaspelin & Luck, 2018b; Gaspelin et al., 2015, 2025; Luck et al., 2021).

More recent accounts of signal suppression emphasize a learning-dependent process that is responsible for the ability to attenuate the priority signal generated by salient stimuli. Two sources of evidence are particularly compelling in this context. The first is a now large body of research demonstrating that physically salient distractors produce reduced attentional interference when they appear in a high-probability location (e.g., Kim & Anderson, 2022; Wang & Theeuwes, 2018), color (e.g., Stilwell et al., 2019; Youn & Anderson, 2024), or shape (Kim et al., 2023; Ogden et al., 2023). It seems that with experience, observers become better at ignoring distracting contexts to which they are frequently exposed and can anticipate experiencing in the future. The second source of evidence is observations that signal suppression in many cases requires prior exposure to the features of a distractor (feature-specific or “first-order” suppression; Gaspelin & Luck, 2018a; Stilwell et al., 2019; Vatterott & Vecera, 2012) along with recent findings demonstrating that initial capture by a salient distractor plays an important role in subsequent signal suppression (Zhang & Gaspelin, 2026).

Findings such those described above have served as inspiration for an updated account of signal suppression that emphasizes the central role of learning-dependent processes or *selection history* underlying the ability of the attention system to reduce or even in some cases eliminate interference from salient distractors (Gaspelin et al., 2025). The precise mechanisms by which selection history gives rise to such gains in distractor rejection, however, remain to be articulated. Anderson et al. (2021) provide a model of attentional control that partitions selection history into three different learning processes capable of shaping attentional priority: associative learning between stimuli and valent outcomes, statistical learning concerning the probability of different sources of perceptual input, and stimulus–response habit learning by which the allocation of attentional priority can be likened to an action that can be triggered by a stimulus to which such prioritization has been repeatedly applied. By hypothesizing three distinct learning-dependent processes that shape the control of attention, the Anderson et al. (2021) model opens up the possibility that more than one underlying mechanism is responsible for experience-dependent reductions in the interference caused by physically salient distractors.

Controversy has arisen concerning the situations under which learning-dependent gains in the ability to ignore salient distractors are feature specific or feature general (see Gaspelin et al., 2025). Facilitated ignoring of high-probability distractors, which has been attributed to statistical learning, is by definition feature specific (Theeuwes et al., 2022), with learned expectations concerning probable distractor contexts down-weighting corresponding sensory input. At the same time, at least when the features of salient distractors are sufficiently variable, suppression of a range of feature-defined distractors has been observed (Drisdelle et al., 2025; Ma & Abrams, 2023a, 2023b; Won et al., 2019). One possibility, articulated in the aforementioned studies, is that learning-dependent gains in distractor rejection can be *either* feature specific *or* feature general depending on the context. Another possibility, tested here, is that such learning-dependent gains can be *both* feature specific *and* feature general, supported by distinct underlying mechanisms capable of operating concurrently, which different experiment contexts can differently engage.

Testing a dual-mechanism account of learning-dependent distractor rejection would be aided by conditions under which feature-specific and feature-general mechanisms make dissociable predictions concerning the manner in which attentional processing is affected by experience. One candidate dissociation can be found in the time course of the learning-dependent effects. Although effects putatively driven by feature-specific statistical learning can persist into a period in which distractor probabilities have been equalized or shifted (e.g., Britton & Anderson, 2020; Wang & Theeuwes, 2020), these effects are apparently short-lived and no longer observable during a second testing session conducted on a different day (Di Caro & Della Libera, 2021), particularly when the distractor and target are defined within different feature dimensions (Sauter et al., 2019). Effects of repeated selection, in contrast, are hypothesized to be much more enduring and robust to extinction (e.g., Jiang, 2018; Jiang & Sisk, 2019; Shiffrin & Schneider, 1977), evident when tested on a later day (e.g., Anderson & Britton, 2019; Anderson et al., 2017; Kim & Anderson, 2019), including with respect to target history effects using an approach very similar to the one used to demonstrate the short-lived nature of distractor probability effects (Di Caro & Della Libera, 2021). How experience repeatedly ignoring a salient distractor influences the magnitude of salience-based attentional capture across multiple testing sessions, however, has not been systematically explored.

In the present study, participants completed a visual search task for a shape-defined target conducted over two testing sessions that occurred on different days. In the first testing session, color singleton distractors were presented under conditions in which distractors were rendered in a specific color and presented at a specific location more frequently than others (high-probability color and location). In the second session, these biased probabilities were removed such that distractors were equiprobable across colors and locations, and a novel distractor color not previously encountered was added to the design. If feature-specific processes supported by statistical learning and feature-general processes supported by the repeated rejection of salient distractors jointly facilitate learning-dependent distractor rejection, we should observe a robust reduction in attentional capture by color singletons appearing in a high-probability color and at a high-probability location in the first session that is no longer evident in the second session (being similar in magnitude even for a novel color with which participants have no prior experience), consistent with a short-lived influence of biased distractor probabilities. The magnitude of attentional capture should also progressively decline across all distractors – even low-probability distractors; in contrast to statistical learning effects, such generalized learning-dependent gains should robustly persist into the second session. Feature-specific gains in distractor rejection that are short lived coupled with feature-general gains that are retained across testing sessions would provide evidence for a dissociation consistent with two distinct learning-dependent processes that together serve to facilitate the ignoring of color singleton distractors.

## Method

### Participants

Twenty-six participants were recruited from the Texas A&M University community. Participants were compensated with course credit. All participants were English-speaking and reported normal or corrected-to-normal visual acuity and normal color vision. All procedures were approved by the Texas A&M University Institutional Review Board. The sample size provided power (1 ˗ β) > 0.99 with α = 0.05 to detect an effect of statistical learning of the magnitude reported in Experiment 1 of Di Caro and Della Libera (2021), which was *d*_*z*_ = 1.495, and power = 0.8 to detect an effect as small as *d*_*z*_ = 0.57 for direct comparisons and η^2^_p_ < 0.075 for all main effects (computed using G*Power 3.1, with the calculation of main effects assuming a modest correlation among repeated measures of 0.5).

### Apparatus

A Dell OptiPlex 7040 equipped with Matlab software and Psychophysics Toolbox extensions (Brainard, 1997) was used to present the stimuli on a Dell P2717H monitor. Responses were entered using a standard US-layout keyboard. The participants viewed the monitor from a distance of approximately 70 cm in a dimly lit room.

### Stimuli

**Fig. 1:**
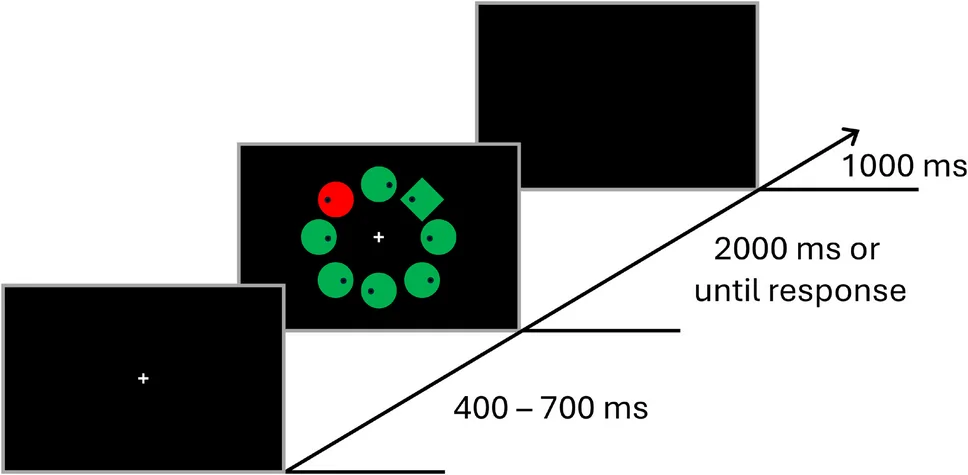
Sequence of events for an example trial. In this trial, the diamond is the target and the red circle is the salient distractor

Each trial consisted of a fixation display, a search display, and an inter-trial interval (ITI). The fixation display consisted of a white plus-sign (0.5° × 0.5°) presented at the center of the screen against a black background (see Fig. 1). The search display consisted of the fixation cross along with eight filled shapes (each approximately 2.9° × 2.9°), each containing a small black dot presented to the left or right of center. The shapes consisted of a single diamond among seven circles or a single circle among seven diamonds, positioned at equal intervals along an imaginary circle with a radius of 7.6°. The colors used for the shapes were an equiluminant red, orange, gold, purple, blue, and green; the target and non-targets were rendered in green, and on distractor-present trials, one of the non-targets (the salient distractor) was rendered in a different color. The ITI consisted of a blank screen. In the event of an incorrect response to the search display or a failure to respond during the presentation of search display, error feedback consisting of the words “Incorrect” or “Too Slow,” presented centrally in white font, was added before the ITI.

### Design

The experiment consisted of eight blocks of 128 trials each (1,024 trials total). Half of the trials in each block were distractor-present trials and half were distractor-absent trials. On each of distractor-present and distractor-absent trials, the target was a circle and diamond equally often, presented at each location on the screen equally often. The dot within the shape was presented on the left and right equally often for the target and was randomly determined for the other seven shapes on each trial with the constraint that half were presented on the left and half on the right.

In the first session, the location of the salient distractor was set to appear in the high-probability location on 45.3% of distractor-present trials and in each of the other seven locations on 7.8% of distractor-present trials. The salient distractor in the first session was rendered in the high-probability color on 53.1% of distractor-present trials and in each of three of the remaining (low-probability) colors on 15.6% of distractor-present trials. In the second session, the salient distractor was presented in each location equally often and in each color near equally often (given the number of distractor colors and the number of trials per block, the distractor was rendered in one of the available colors one trial less frequently than the others, randomly determined each block), with an additional color (the novel color) added into the design. The order of trials in each session was random.

The high-probability location and color in the first session were determined by participant number, cycling through the different possibilities every eight and five participants, respectively, ensuring that different combinations of high-probability location and color were used. Which color served as the novel color, appearing only in the second session, was tied to the high-probability color such that specific color pairs were used to ensure robust discriminability (blue-red, purple-orange, gold-purple, orange-blue, red-gold), with each color serving once as the high-probability distractor color and once as the novel color every five participants.

### Procedure

The first and second session were scheduled 1–2 days apart. Participants were instructed to find the target and report the side of the dot within the target as quickly and accurately as possible. They were informed that the uniquely colored item would never be the target and that they could simply ignore the color singleton distractor. The first session began with 30 practice trials to familiarize participants with the task, while the second session began immediately with the experiment trials following a reminder of task instructions.

Each trial began with the fixation display that randomly varied in duration from 400 to 700 ms, followed by the search array, which remained on-screen until participants indicated a response or the trial timed out after 2,000 ms. Following a correct response, the search array was followed by a blank 1,000-ms ITI; in the event of an erroneous response or a failure to respond before the timeout, a 1,000-ms feedback display was inserted after the search array and before the ITI. Participants pressed the “N” key if the dot within the target was on the left and the “M” key if the dot within the target was on the right. A minimum 30-s break was inserted between each block of trials, with participants needing to manually resume the experiment with a keypress when ready.

### Data analysis

Only participants who contributed data for both sessions were included in analyses. Response times (RTs) faster than 150 ms and exceeding three *SD*s of the conditional mean of each session were trimmed. Only RTs for correct trials were retained for analysis. Distractor-present trials in the first session were binned according to the combination of color and location probability (location/color: low/low, high/low, low/high, high/high). Distractor-present trials in the second session were binned according to the location and color probabilities that were present in the prior session, with an additional category for novel color distractors. For the second session, in the event of null effects of biased location and color probabilities from the first session, Bayesian statistics were computed in order to quantify the probability of the null hypothesis (JASP 18.3.0 using default priors).

The effect of experience rejecting salient distractors on attentional capture was computed by comparing the low/low distractor condition to distractor-absent trials, both across epochs within the first session and across sessions. The effects of statistical learning were computed via a 2 × 2 analysis of variance (ANOVA) across distractor-present conditions, with (previous) location (high/low) and color (high/low) probability as factors. The effect of novelty in the second session was computed by comparing novel-color distractor trials to trials on which the distractor was rendered in a previously low-probability color. To ensure an adequate number of trials per bin across all conditions, epochs were defined as pairs of blocks (e.g., the first epoch consisted of the first and second block, the second epoch the third and fourth block); even so, when computing the effects of statistical learning for each epoch, trials on which the distractor was rendered in the previously high-probability color and location on the same trial (high/high) in the second session happened infrequently (so much so that such trials did not occur at all for some epochs for some participants), and so these trials were not included in analyses computing the effects of statistical learning over epoch. This reflects a limitation inherent to the design of the second session, with equalizing the probability of different distractor colors and locations necessitating that the confluence of the previously high-probability color and location occur infrequently, precluding more fine-grained assessment of the time course of this particularly sensitive condition and/or a full factorial analysis when binning trials by epoch.

## Results

### First session

**Fig. 2:**
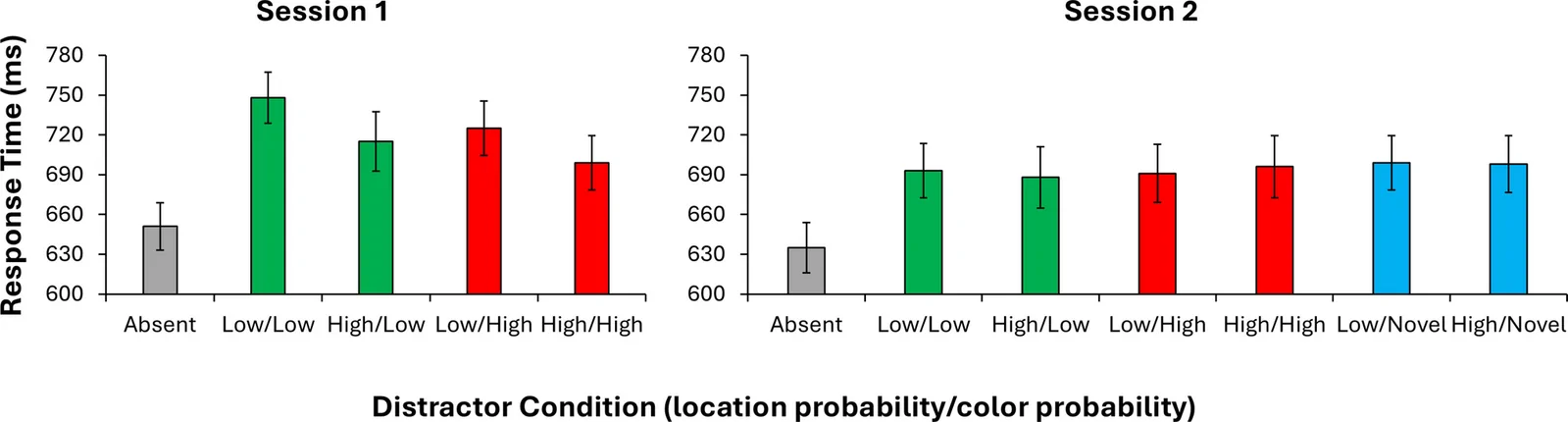
Manual response time as a function of distractor condition ([previous] location/color probability) in the first and second session of the experiment. Error bars represent the *SEM*

**Fig. 3:**
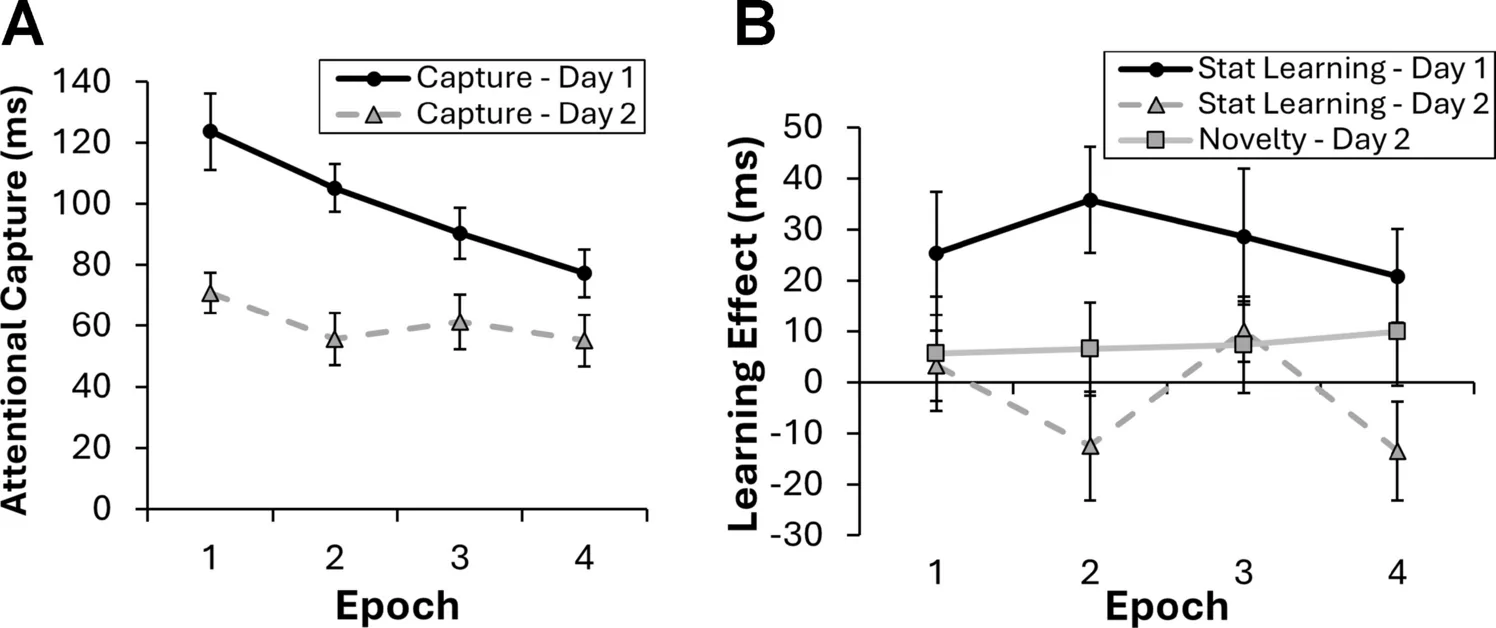
Attention capture effects (**A**) and the effects of statistical learning and novelty (**B**) on attentional capture across epoch for each of the two sessions/days of testing. Capture reflects the difference between (previously) low-probability distractor and distractor-absent trials. Stat(istical) learning reflects the mean of the difference between distractor-present trials when the distractor was presented in the (previously) high- vs. low-probability location (location effect) and high- vs. low-probability color (color effect). Novelty reflects the difference in mean response time when the distractor was rendered in the novel color vs. a previously low-probability color specifically in Experiment 2 (when the novel color was introduced). Error bars represent the *SEM*

The RT was slowed on low-probability (with respect to both color and location) distractor trials compared to distractor-absent trials, *t*(25) = 13.78, *p* <.001, *d*_*z*_ = 2.70, indicative of attentional capture (Fig. 2). The magnitude of this distraction effect decreased over epoch, being characterized by a significant linear trend, *F*(1,25) = 19.69, *p* <.001, η^2^_p_ =.441 (Fig. 3A). On distractor-present trials, there was a main effect of both color, *F*(1,25) = 16.36, *p* <.001, η^2^_p_ =.396, and location probability, *F*(1,25) = 44.02, *p* <.001, η^2^_p_ =.638, with RT being reduced when the distractor appeared in a high-probability color and location. Consistent with prior reports (e.g., Kim & Anderson, 2022; Wang et al., 2019), the influence of biased color and location probabilities was evident even during the first epoch of trials, *t*(25) = 2.09, *p* =.047, *d*_*z*_ = 0.41, being rapidly learned (Fig. 3B). The interaction between color and location was not significant, *F*(1,25) = 0.33, *p* =.569 (Fig. 2), with the two main effects producing additive influences on performance, and the influence of color and location probabilities was strongly correlated across participants, *r* =.674, *p* <.001. The same analyses performed on accuracy revealed higher accuracy on distractor-absent trials, *t*(25) = 3.27, *p* =.003, *d*_*z*_ = 0.64, with no significant main effects or interaction on distractor-present trials, *F*s < 1.33, *p*s >.25 (Table 1), ruling out any potential contamination from a speed-accuracy tradeoff in the observed RT effects.

**Table 1:** Mean accuracy (percent correct) as a function of distractor condition in the first and second session of the experiment. The *SEM* is indicated in parentheses

	Distractor condition						
	Absent	Low/Low	High/Low	Low/High	High/High	Low/Novel	High/Novel
Session 1	97.4 (0.48)	96.2 (0.67)	96.4 (0.77)	96.5 (0.55)	96.5 (0.89)	–-	–-
Session 2	97.0 (1.13)	95.9 (1.23)	96.5 (1.28)	95.9 (1.41)	96.0 (1.33)	96.5 (1.24)	97.0 (1.74)

### Second session

The RT was again slowed on low-probability distractor trials compared to distractor-absent trials, *t*(25) = 10.15, *p* <.001, *d*_*z*_ = 1.99 (Fig. 2); this slowing was significantly reduced compared to that observed in the first session, *t*(25) = 5.61, *p* <.001, *d*_*z*_ = 1.10, with attentional capture being reduced by approximately 40% across sessions (Fig. 3A). Accuracy was also significantly higher on distractor-absent trials, *t*(25) = 3.92, *p* <.001, *d*_*z*_ = 0.77 (Table 1).

The effect of the prior distractor probabilities was no longer evident in RT, with no main effect of either color, *F*(1,25) = 0.16, *p* =.691, or location, *F*(1,25) < 0.01, *p* =.999, nor an interaction, *F*(1,25) = 0.33, *p* =.572 (Fig. 2). A Bayesian ANOVA favored a model with no main effects or interaction, BF_M_ = 6.38, with the next-best model, characterized by a main effect of location, being comparatively unlikely, BF_10_ = 0.27. The effect of the (previously) biased probabilities was also significantly larger in the first session compared to the second session, *t*(25) = 4.47, *p* <.001, *d*_*z*_ = 0.88.^1^ There was also no evidence for an effect of the previous high-probability location, *t*(25) < 0.01, *p* =.999, BF_01_ = 4.83, or color, *t*(25) = 0.80, *p* =.432, BF_01_ = 3.61, when analyses were restricted to the first epoch of trials (Fig. 3B). RT was numerically slower on novel compared to previously low-probability color distractor trials, but this difference was not significant, *t*(25) = 1.04, *p* =.307, with a Bayesian t-test providing anecdotal evidence for the null hypothesis, BF_01_ = 2.96; this was also true when restricting analysis to the first epoch of trials, *t*(25) = 0.50, *p* =.619 (Fig. 3B), in this case with a Bayesian t-test providing moderate evidence in favor of the null hypothesis, BF_01_ = 4.30.

The same ANOVA performed over accuracy on distractor-present trials also revealed no main effects or interaction, *F*s < 0.45, *p*s >.50. There was also no significant difference between novel compared to previous low-probability color distractor trials, *t*(25) = 1.21, *p* =.239 (Table 1), with the direction of difference being opposite the non-significant effect observed in RT, further suggesting that there was no residual influence of statistical learning on attentional processing.

## Discussion

The present study tested a dual-mechanisms account by which feature-specific and feature-general mechanisms of learning-dependent distractor rejection jointly contribute to experience-dependent reductions in attentional capture by physically salient stimuli. Consistent with a feature-specific mechanism, participants were facilitated in ignoring distractors rendered in a high-probability color and appearing in a high-probability location during the first testing session. These effects of statistical learning were short-lived, no longer evident when assessed 1 or 2 days later in a context in which the biased probabilities had been removed, consistent with prior reports (Di Caro & Della Libera, 2021; Sauter et al., 2019). At the same time, attentional capture by low-probability distractors, which would not be subject to benefits from statistical learning, nevertheless progressively declined with experience in the first session, consistent with a feature-general influence built up from experience rejecting salient distractors. Importantly, this learning-dependent benefit persisted for the entire duration of the second session, with attentional capture significantly reduced compared to the prior session. This dissociation in the time course of learning-dependent effects suggests that two distinct attentional processes contribute to the ability to more efficiently ignore salient stimuli as a function of selection history.

The findings of the present study have important implications for how signal suppression is conceptualized. Often discussed as a single, unitary process (e.g., Gaspelin et al., 2025; Luck et al., 2021), signal suppression – and learning-dependent influences on the processing of salient distractors more broadly – might instead reflect more than one component process, each of which likely operates via unique principles. Understanding when and to what degree the magnitude of attentional capture by salient stimuli is impacted by experience, and the underlying processes responsible for this impact, will be incomplete when only one influence of selection history is considered. For example, debate has arisen concerning whether signal suppression is feature specific or can generalize across features (see Gaspelin et al., 2025, for a review); the present study suggests that both of these possibilities could be simultaneously true, with statistical learning influencing feature-specific distractor processing and protracted experience rejecting salient distractors influencing the processing of salient signals more generally.

Theories of selection history posit multiple distinct mechanisms by which learning history shapes attentional priority (for reviews, see Anderson, 2024; Anderson et al., 2021). As recent accounts of signal suppression emphasize experience-dependent processes (e.g., Gaspelin & Luck, 2018b; Gaspelin et al., 2025; Luck et al., 2021), it is perhaps unsurprising that more than one of the mechanisms that collectively comprise selection history would be at play in shaping attentional priority for stimuli that are repeatedly experienced as task irrelevant. Although future research is needed to more fully characterize the learning-dependent mechanisms underlying the ability to ignore salient distractors and to what degree they overlap with the mechanisms of statistical learning and habit learning articulated in Anderson et al. (2021), the observations of the present study can be accounted for by appealing to this selection history framework.

The present study conceptually replicates Sauter et al. (2019) and Di Caro and Della Libera (2021) with respect to the effect of location probabilities between sessions, and extends these findings to biased color probabilities. Even in the first epoch of trials within the second testing session, the improved ignoring of high-probability distractors was no longer evident when tested on a different day. This contrasts with findings that the effects of statistical learning on attentional priority can extend into extinction (e.g., Britton & Anderson, 2020; Kim & Anderson, 2021; Ogden et al., 2023) or periods of probability reversal (Wang & Theeuwes, 2020), suggesting that the gap between sessions was operative in extinguishing these learning-dependent gains. This is not to imply that feature-specific influences of statistical learning on the processing of salient distractors are inherently short-lived, as more enduring influences have been observed when the target and distractor are defined within the same feature dimension (Sauter et al., 2019); however, it is clear that the feature-specific influences that were observed in the present study have qualitatively different temporal profiles than concurrent feature-general influences, suggesting dissociable influences on distractor processing.

The present study identified a highly robust effect that is seldom discussed in the attentional capture literature by which the magnitude of attentional capture by a physically salient stimulus progressively decreases with experience rejecting it as a non-target, which was evident even for low-probability distractors. In this respect, ignoring salient distractors can be thought of as a learned skill that improves with practice, with a persistence consistent with other kinds of skill learning, including attentional prioritization of stimuli that consistently serve as a target of search (Anderson & Britton, 2019; Anderson et al., 2017; Kim & Anderson, 2019). This kind of feature-general learning-dependent de-prioritization may be related to the habituation of the orienting response to salient stimuli (Turatto, 2023). It may also be related to situations in which participants can learn to ignore salient signals within an entire feature dimension, given that the present study used color singleton distractors in the context of a shape-defined target (see Sauter et al., 2019).

Even an entirely novel color not previously encountered in the first session captured attention to a similar degree when compared to distractors rendered in the previously most frequently encountered color. Coupled with a generalized reduction in the magnitude of attentional capture by physically salient stimuli in the absence of residual probability effects, this finding provides converging evidence for a feature-general influence on distractor processing. To the degree that experience rejecting a salient stimulus gradually improves ignoring of the specific rejected stimulus as an exemplar, a substantial difference between the magnitude of interference caused by high-probability distractors (which participants had rejected many times) and novel distractors would have been observed; this was clearly not the case, with all salient distractors slowing RT to a similar degree that was facilitated relative to the prior testing session.

Consistent with the effects of statistical learning in the context of search guidance (Anderson, 2026), feature-based and location-based learning produced additive effects on attentional prioritization in the present study. Such additivity has also been observed in the context of effects of statistical learning and associative learning between stimuli and valent outcomes (Kim & Anderson, 2021; Ogden et al., 2023), and suggests that distinct underlying processes contribute to the probability-dependent improvement in ignoring with experience. The present study therefore suggests that, in addition to the distinction between feature-specific and feature-general contributions to the ability to more efficiently ignore salient distractors with learning, the feature-specific contribution may further decompose into separable color- and location-based component processes. Interestingly, it was also the case that participants who were the most influenced by the color probabilities also tended to be the most influenced by the location probabilities, suggesting that while recent findings support additive contributions from color-based and location-based learning (see also Anderson, 2026; Kim & Anderson, 2021), some participants may be generally more sensitive to the statistics of the task than others.

One limitation of the present study is that the nature of distractor processing was not assessed directly using eye tracking or electrophysiological measures. It is unclear whether the observed learning-dependent reductions in distractor inference reflect improvements in proactive suppression or facilitated disengagement following attentional capture (reactive suppression). In fact, it is possible that this distinction could in part explain the observed dissociation, with feature-specific mechanisms of distractor suppression being proactive and feature-general mechanisms being reactive in nature or vice versa. Future studies should seek to measure distractor processing more directly under conditions similar to those tested here to provide additional mechanistic insights.

In conclusion, the present study supports the idea that the ability to more efficiently ignore salient distractors with learning is the product of both feature-specific and feature-general mechanisms. Observers become better able to ignore frequently encountered distractors and become better able to ignore distracting stimuli in general with experience. While the effects of stimulus frequency were short-lived, the effects of repeatedly ignoring salient stimuli were more enduring, with the distinct time courses evident in the present study supporting a dissociation between them. Attempts to explain the effects of selection history on attentional processing of physically salient stimuli should therefore be sensitive to the possibility that more than one learning-dependent process is contributing to attentional performance.

## Data Availability

Neither the data nor the materials have been made available on a permanent third-party archive; requests for the data or materials can be sent via email to brian.anderson@tamu.edu.

## Footnotes

1 There is a potential asymmetry in statistical power for the assessment of reduced attentional capture by low-probability distractors and a reduced influence of statistical learning across sessions, as the former was pooled over a greater number of trials. However, the significant reduction in attentional capture by low-probability distractors across sessions remains when sub-sampling trials to the condition with the fewest number of trials in the assessment of statistical learning, *t*(25) = 4.05, *p* <.001, and the reduced influence of statistical learning across sessions is retained when the effect of statistical learning is computed as a proportion of overall attentional capture (which is reduced in the second session), *t*(25) = 2.27, *p* =.032.

## Code availability

Analysis code has not been made available on a permanent third-party archive; requests for the analysis scripts used can be sent via email to brian.anderson@tamu.edu.

## Declarations

### Open practices statement

The experiment reported in this article was not formally preregistered. Neither the data nor the materials have been made available on a permanent third-party archive; requests for the data or materials can be sent via email to brian.anderson@tamu.edu.
